# Ultrasound assessment of carpal tunnel in rheumatoid arthritis and idiopathic carpal tunnel syndrome

**DOI:** 10.1007/s10067-020-05293-z

**Published:** 2020-07-21

**Authors:** Gianluca Smerilli, Andrea Di Matteo, Edoardo Cipolletta, Sergio Carloni, Antonella Incorvaia, Marco Di Carlo, Walter Grassi, Emilio Filippucci

**Affiliations:** 1 Rheumatology Unit, Department of Clinical and Molecular Sciences, “Carlo Urbani” Hospital, Polytechnic University of Marche, Via Aldo Moro 25, 60035 Jesi, Italy; 2 Leeds Institute of Rheumatic and Musculoskeletal Medicine, University of Leeds, Leeds, UK; 3 Orthopaedic Unit, “Carlo Urbani” Hospital, Via Aldo Moro 25, Jesi, Italy

**Keywords:** Carpal tunnel syndrome, Nerve compression syndromes, Rheumatoid arthritis, Ultrasonography

## Abstract

**Objectives:**

To comparatively assess the sonographic spectrum of carpal tunnel syndrome (CTS) in patients with rheumatoid arthritis (RA) and in patients with idiopathic CTS.

**Methods:**

Fifty-seven RA patients and 25 idiopathic CTS patients were consecutively enrolled. The diagnosis of CTS in RA patients was made according to clinical history and examination. The following sonographic findings were assessed at carpal tunnel level: median nerve cross-sectional area (CSA) at the carpal tunnel proximal inlet, finger flexor tendons tenosynovitis, radio-carpal synovitis and intraneural power Doppler (PD) signal.

**Results:**

CTS was diagnosed in 15/57 RA patients (26.3%). Twenty-three RA wrists with CTS, 84 RA wrists without CTS and 34 idiopathic CTS wrists were evaluated. The average CSA of the median nerve was higher in idiopathic CTS than in RA wrists with CTS (17.7 mm^2^ vs 10.6 mm^2^, *p* < 0.01). A higher rate of inflammation of synovial structures (flexor tendons sheath and/or radio-carpal joint) was found in RA wrists with CTS compared with those without CTS (*p* = 0.04) and idiopathic CTS (*p* = 0.02). Intraneural PD signal was more common in CTS (in both RA and idiopathic CTS) wrists compared with wrists without CTS (*p* < 0.01).

**Conclusion:**

The sonographic spectrum of CTS in RA patients is characterized by an inflammatory pattern, defined by the presence of finger flexor tendons tenosynovitis and/or radio-carpal joint synovitis. Conversely, a marked median nerve swelling is the dominant feature in idiopathic CTS. Intraneural PD signal is a frequent finding in both conditions.

**Table:** 

**Key Points** • *Carpal tunnel syndrome (CTS) associated with rheumatoid arthritis (RA) and idiopathic CTS have distinct ultrasound patterns.* • *The most characteristic sonographic features of CTS in RA patients are those indicative of synovial tissue inflammation at carpal tunnel level. Conversely, marked median nerve swelling is the dominant finding in idiopathic CTS.* • *Intraneural power Doppler signal is a frequent finding in both conditions.* • *In patients with CTS, differently from electrophysiology, US can provide clues prompting a rheumatology referral in case of prominent inflammatory findings at carpal tunnel level.*

## Introduction

Carpal tunnel syndrome (CTS) is the most common entrapment neuropathy [1]. CTS can be associated with several rheumatologic disorders, most commonly rheumatoid arthritis (RA) [2, 3]. Idiopathic CTS is characterized by the absence of underlying identifiable conditions [4].

The diagnosis of CTS is based on clinical history and physical examination. Nerve conduction studies and ultrasound (US) can provide further clinically relevant information [5–10]. Three meta-analysis confirmed that US is helpful for the diagnosis of CTS [11–13]. In 2012, American Association of Neuromuscular and Electrodiagnostic Medicine evidence-based guidelines affirmed that US adds value to electrodiagnosis [14]. In fact, US may identify several causes of median nerve entrapment at carpal tunnel level (e.g. flexor tendon tenosynovitis, wrist synovitis, or crystal deposits).

The most referenced US finding that characterizes idiopathic CTS is median nerve swelling, evaluated measuring its cross-sectional area at the proximal inlet of carpal tunnel [15–17]. More recently, a few studies noted that intraneural power Doppler (PD) signal is common in CTS [18, 19] and may predict better surgical outcomes [20].

However, despite a growing body of literature confirming the diagnostic potential of US in idiopathic CTS, only two studies have investigated the role of US in the assessment of CTS in patients with RA [21, 22].

The main aim of this study was to assess the spectrum of sonographic findings at carpal tunnel level in a cohort of CTS patients with RA and to compare these findings with those observed in patients with idiopathic CTS.

## Materials and methods

### Patients

Consecutive patients from the Rheumatology Unit of “Carlo Urbani” Hospital, in Jesi (Ancona, Italy), fulfilling ACR/EULAR 2010 RA classification criteria were enrolled. Consecutive patients from the Rheumatology and Orthopaedic Units of the same hospital with a clinical diagnosis of idiopathic CTS according to the American Academy of Neurology practice parameter [23] were recruited as controls. Exclusion criteria were age < 18 years and previous surgical decompression of carpal tunnel.

The study was conducted in accordance with the Helsinki Declaration and was approved by the local ethics committee (Comitato Etico Regione Marche, number 262). All patients signed informed consent.

### Clinical assessment

The following data were registered for each RA patient: age, sex, rheumatoid factor (RF), anti-citrullinated protein antibodies (ACPA), disease duration, erythrocyte sedimentation rate (ESR), C-reactive protein (CRP), Clinical Disease Activity Index (CDAI) and treatment. The neuropathic pain features in RA patients were investigated through the PainDETECT questionnaire, a tool with proven validity in patients with chronic inflammatory arthritis [24, 25]. The diagnosis of CTS followed a thorough clinical history and physical examination and was made according to the American Academy of Neurology practice parameter for CTS [23]. If present, the severity of CTS was graded using historical-objective scale (Hi-Ob scale), a validated measurement tool based on clinical examination [26]. According to this scale CTS severity was graded into five stages: stage 1, only nocturnal paresthesia; stage 2, diurnal paresthesia; stage 3, sensory deficit; stage 4, hypotrophy and/or motor deficit of median innervated thenar muscles; and stage 5, complete atrophy or plegia of median innervated thenar muscles.

### Ultrasound assessment

Sonographic evaluation was performed by a rheumatologist blinded to the clinical data. The US assessment was carried out using a MyLab Class C (Esaote SpA, Genoa, Italy) US system working with a 6–18 MHz linear probe. Patients were seated in a comfortable position, with the forearm resting supine on the examination bed and fingers in neutral position. The power Doppler (PD) frequency was set at 9.1 MHz with a pulse repetition frequency of 750 Hz. Each wrist was scanned in both longitudinal and transverse views as indicated by the 2017 EULAR standardized procedures for US imaging in rheumatology [27].

The following US pathologic findings were assessed at the carpal tunnel level: median nerve swelling measuring its cross-sectional area (CSA) at the carpal tunnel proximal inlet (at the level of the pisiform bone) by tracing a continuous line within the hyperechogenic boundary of the nerve, finger flexor tendons tenosynovitis and radio-carpal joint synovitis. Tenosynovitis was defined as abnormal anechoic and/or hypoechoic tendon sheath widening according to Outcome Measures in Rheumatology (OMERACT) definition [28]. The presence of a bifid median nerve was recorded.

The intraneural PD signal was scored as present/absent and semi-quantitatively according to the following grading system: 0 = no PD signal, 1 = 1 single vessel within median nerve, 2 = 2 or 3 single or 2 confluent vessels and 3 = more than 3 single or more than 2 confluent vessels [20, 29].

A thorough US examination with longitudinal and transverse scans was conducted to distinguish intraneural PD signal from the presence of a persistent median artery.

For the analysis, RA wrists were divided in two groups: CTS+ RA wrists (i.e. wrists with CTS in RA patients) and CTS- RA wrists (i.e. wrists without CTS in RA patients, excluding those belonging to RA patients presenting CTS symptoms in the contralateral wrist).

### Statistical analysis

Results are expressed as mean ± standard deviation (SD) for quantitative variables with a normal distribution, as median and interquartile range (IQR) for quantitative variables with a non-normal distribution and as number and/or percentage for qualitative variables. Quantitative variables were tested for normality using Kolmogorov–Smirnov test. One-way analysis of variance (ANOVA) was used for quantitative variables; chi-square test was used for qualitative variables (Bonferroni correction was applied to correct for multiple comparisons). The association between the presence of clinical diagnosis of CTS and continuous variables was tested using point biserial correlation, while for dichotomous variables chi-squared test and Cramer’s V were used. Statistical significance was set at *p* values of less than 0.05. The statistical analysis was performed using SPSS version 23 statistical software (SPSS, Inc., Chicago, IL, USA).

## Results

A total of 57 RA patients and 25 patients with idiopathic CTS were consecutively enrolled in this study. CTS was diagnosed in 15 out of 57 RA patients (26.3%). RA patients’ demographic and clinical characteristics are reported in Table 1. Patients with idiopathic CTS had a mean age of 63.3 ± 13.7 years and their male-to-female ratio was 1:1.27.

**Table 1:** Rheumatoid arthritis patients’ demographic and clinical characteristics

	RA patients with CTS	RA patients without CTS	Total of RA patients
Number of patients	15	42	57
Age (years)	59 ± 11	55 ± 14	57 ± 13
Gender (M/F)	1/14	4/38	5/52
Disease duration (RA)	14 ± 11	14 ± 10	14 ± 10
RF positivity (%)	71.4	66.6	68.6
ACPA positivity (%)	78.6	66.6	70.6
ESR (mm/first hour)	24 ± 24	23 ± 19	23 ± 20
CRP (mg/dL)	1 ± 1	0.8 ± 0.9	0.8 ± 1
CDAI	18.5 ± 12.0	10.0 ± 11.0	12 ± 12
Remission (≤ 2.8)	1 (6.6%)	18 (43%)	19 (33.3%)
Low disease activity (2, 9, 10)	4 (26.6%)	8 (19%)	12 (21%)
Moderate disease activity (10, 1–20)	4 (26.6%)	8 (19%)	12 (21%)
High disease activity (> 22)	6 (40%)	8 (19%)	14 (24.5%)
MTX monotherapy (%)	21.4	14	14.3
MTX + biologic DMARD (%)	50	26	32
Biologic DMARD monotherapy (%)	7	43	34

The values are the mean ± SD, unless stated otherwise

*Abbreviations*: *ACPA* anti-citrullinated protein antibodies, *CDAI* Clinical Disease Activity Index, *CRP* C reactive protein, *CTS* carpal tunnel syndrome, *ESR* erythrocyte sedimentation rate, *DMARD* disease-modifying antirheumatic drugs, *F* female, *M* male, *MTX* methotrexate, *RA* rheumatoid arthritis, *RF* rheumatoid factor

**Fig. 1:**
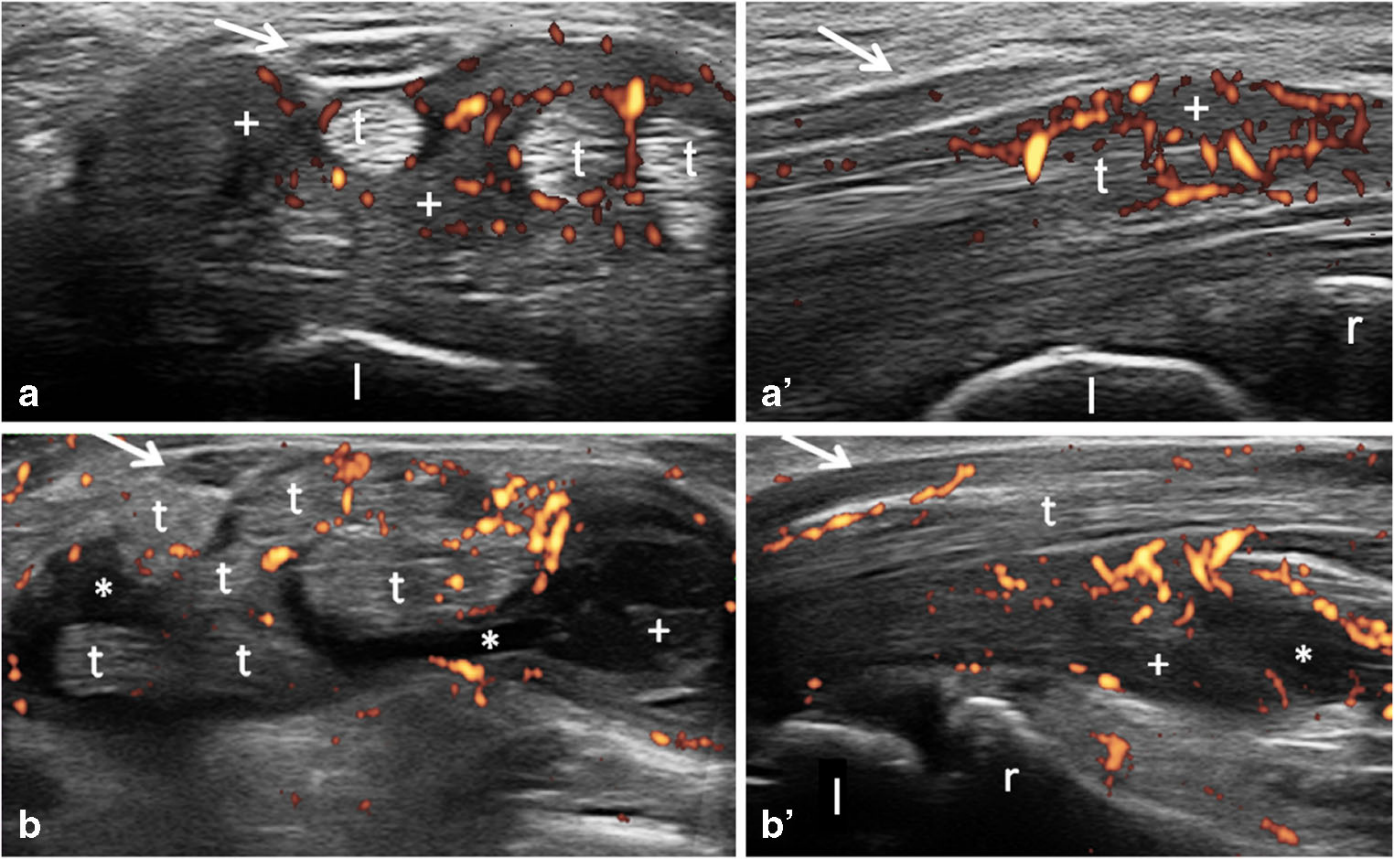
Rheumatoid arthritis. Carpal tunnel syndrome. Representative ultrasound images obtained in transverse (a and b) and longitudinal (a′ and b′) scans showing tenosynovitis of finger flexor tendons. Note the abnormal tendon sheath widening due to inflamed areas of synovial hypertrophy (plus signs) with evident power Doppler signal and effusion (asterisks) and no relevant morphostructural changes of the median nerve (arrows). The image in b was acquired slightly proximally to carpal tunnel inlet in order to better depict the inflammatory abnormalities. r = radius, l = lunate bone, t *=* finger flexor tendons

Clinical diagnosis of CTS in RA patients was made in 23 wrists out of 114 (20.2%). Twenty-three CTS+ RA wrists (i.e. wrists with CTS in RA patients), 84 CTS- RA wrists (i.e. wrists without CTS in RA patients, excluding those belonging to RA patients presenting CTS symptoms in the contralateral wrist) and 34 idiopathic CTS wrists were evaluated (Fig. 1).

**Fig. 2:**
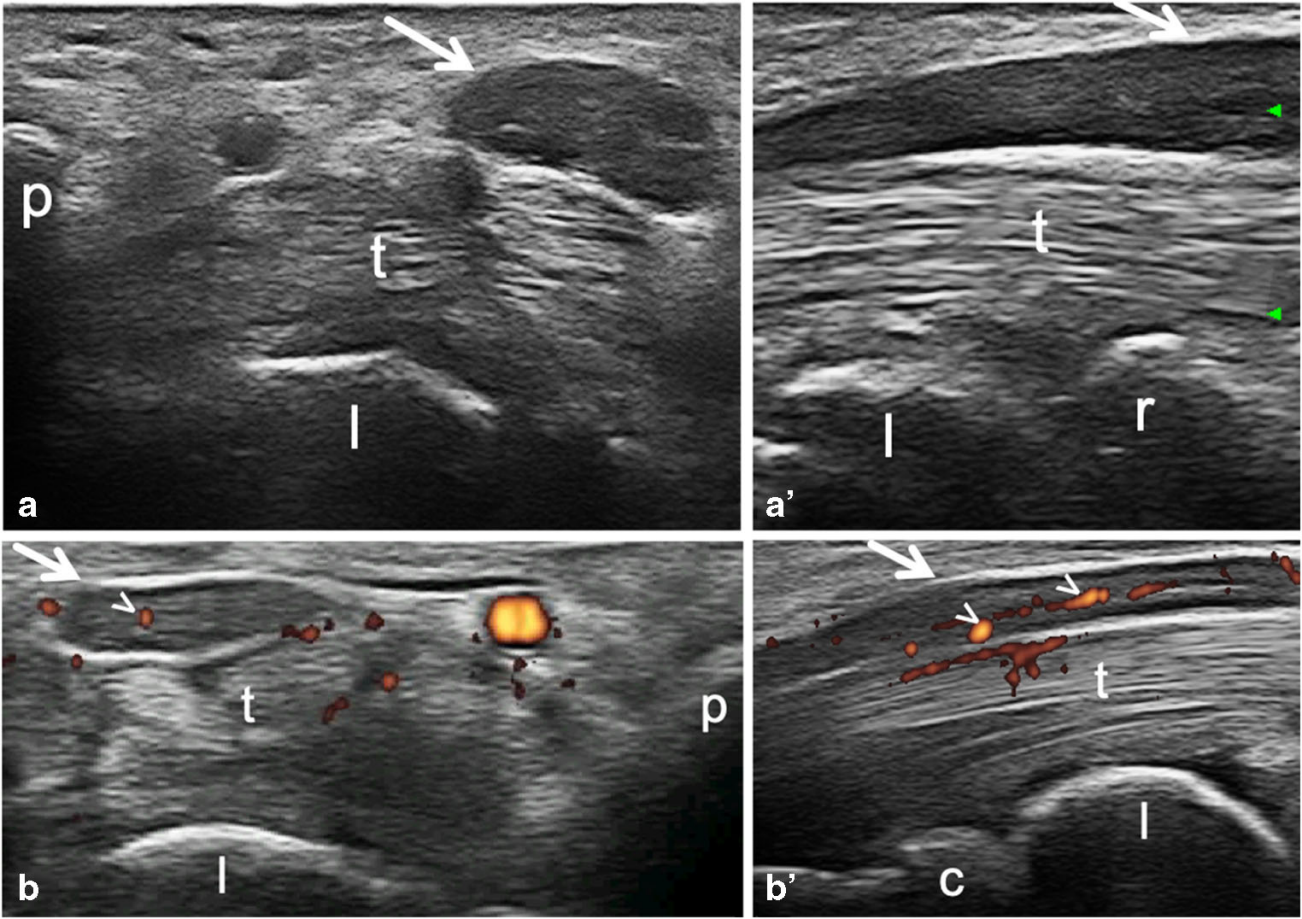
Idiopathic carpal tunnel syndrome. Representative images obtained with transverse (a and b) and longitudinal (a′ and b′) scans. Transverse scans were conducted at the carpal tunnel proximal inlet. (a–a′) B-mode US examination of a 74-year-old female patient showed median nerve marked enlargement (arrows). (b–b′) Power Doppler US examination of a 52-year-old male patient showed abnormal intraneural blood flow (arrowheads), and mild swelling of the median nerve (arrows). c *=* capitate bone, p *=* pisiform bone, r = radius, l = lunate bone, t *=* finger flexor tendons

Table 2 shows US findings from CTS+ RA wrists, CTS- RA wrists, and idiopathic CTS controls. The average CSA of the median nerve was statistically different between the 3 groups (CTS+ RA wrists vs CTS- RA wrists, *p* = 0.02; CTS+ RA wrists vs idiopathic CTS, *p* < 0.01; CTS- RA wrists vs idiopathic CTS, *p* < 0.01) (Fig. 2).

**Table 2:** Ultrasound findings in CTS+ RA wrists, CTS− RA wrists and idiopathic CTS wrists

	CTS+ RA	CTS- RA	Idiopathic CTS
Number of wrists	23	84	34
Median nerve CSA (mm^2^)	10.6 ± 4.2	8.6 ± 2.1	17.7 ± 4.5
Tenosynovitis of finger flexor tendons, *n* (%)	6 (26.1%)	8 (9.5%)	4 (11.8%)
Synovitis of radio-carpal joint, *n* (%)	4 (17.4%)	10 (11.9%)	1 (2.9%)
Tenosynovitis of finger flexor tendons and/or synovitis of radio-carpal joint, *n* (%)	9 (39.1%)	13 (15.4%)	4 (11.8%)
Presence of intraneural PD signal, *n* (%)	10 (43.5%)	12 (14.3%)	22 (64.7%)
Bifid median nerve, *n* (%)	1 (4.3%)	3 (3.5%)	3 (8.8%)

The values are the mean ± SD, unless stated otherwise

*Abbreviations*: *CSA* cross sectional area at the proximal inlet of the carpal tunnel; *CTS* carpal tunnel syndrome; *CTS+ RA wrists* wrists with CTS in RA patients; *CTS− RA wrists* wrists without CTS in RA patients, excluding those belonging to RA patients presenting CTS symptoms in the contralateral wrist; *PD* power Doppler; *RA* rheumatoid arthritis

A higher rate of inflammatory findings at carpal tunnel level (i.e. finger flexor tendons tenosynovitis and/or radio-carpal joint synovitis) was found in RA wrists with CTS compared with idiopathic CTS (39.1% vs 11.7%, *p* = 0.02) and RA wrists without CTS (39.1% vs 15.4%, *p* = 0.04).

Intraneural PD signal was a relatively frequent finding in both CTS+ RA wrists and idiopathic CTS, without a statistical significant difference between these groups (*p* = 0.11). Of note, its presence was more common in CTS wrists (in both RA and idiopathic CTS) compared with wrists without CTS (*p* < 0.01).

Higher CDAI (18.5 ± 12.0 vs 10.0 ± 11.0) and painDETECT questionnaire score (7.2 ± 7.2 vs 4.8 ± 5.5) were reported in RA patients with CTS compared with RA patients without CTS (*p* = 0.02 and *p* < 0.01). No statistical difference was found in the severity of CTS measured according to Hi-Ob (Table 3) scale between CTS in RA patients and idiopathic CTS (*p* = 0.86).

**Table 3:** Severity of carpal tunnel syndrome according to Hi-Ob scale

	Stage 1	Stage 2	Stage 3	Stage 4	Stage 5
CTS+ RA wrists, *n* (%)	5 (21.7%)	3 (13.0%)	10 (43.5%)	5 (21.7%)	0
Idiopathic CTS wrists, *n* (%)	4 (11.8%)	10 (29.4%)	12 (35.3%)	8 (23.5%)	0

*Abbreviations*: *CTS* carpal tunnel syndrome, *RA* rheumatoid arthritis

## Discussion

CTS is a constellation of signs and symptoms, which can be sustained by different pathogenetic mechanisms, all converging towards the compression of the median nerve [30–32].

CTS is frequent in RA patients and is mainly related to the inflammatory process [33, 34]. On the other hand, inflammation is characteristically absent in idiopathic CTS [35, 36].

The aim of our study was to comparatively assess the sonographic spectrum of CTS in patients with RA and in patients with idiopathic CTS.

Our data demonstrate that CTS associated with RA and idiopathic CTS have distinct US patterns.

The most characteristic sonographic features of CTS in RA patients were those indicative of synovial tissue inflammation at carpal tunnel level (i.e. finger flexor tendons tenosynovitis and/or radio-carpal joint synovitis). Conversely, marked median nerve swelling was the dominant finding in idiopathic CTS. A consensus on the appropriate CSA cut-off value for median nerve pathologic swelling has not been reached yet, ranging from 6.5 to 15 mm^2^ [37]. This may be due to several factors, including the operator dependency, different scanning protocols, and various US systems. Of note, in our cohort of RA patients, the US diagnosis of CTS would have been missed in 17 out of 23 wrists (73.9%) using 12 mm^2^ as the CSA cut-off value, which is considered to be highly specific [38]. In the same group of patients, the diagnosis would have been missed in 13 out of 23 wrists (56.5%) using the more sensitive threshold of 10 mm^2^ [39]. On the other hand, in idiopathic CTS, the cut-offs of 12 mm^2^ and 10 mm^2^ would have led to miss the diagnosis only in 2 out of 34 wrists (5.8%) and in 1 out 34 wrists (2.9%), respectively.

To the best of our knowledge, the present study was the first to comparatively assess the prevalence of intraneural PD signal in RA patients with CTS, RA patients without CTS, and patients with idiopathic CTS. The presence of such finding was frequent in RA patients with CTS as well as in those with idiopathic CTS, without a statistically significant difference between these two groups. Conversely, in RA patients, a significantly higher prevalence of intraneural PD signal has been detected in those with CTS compared with those without CTS.

Thus, our results confirm the role of intraneural PD signal as a biomarker of median nerve compression [29], even if its diagnostic value is still a matter of debate [37].

In a study carried out by Karadag et al. [22], consecutive RA patients were included and a diagnosis of CTS was made if one of the following criteria were satisfied: symptoms of CTS plus US measured CSA of median nerve >13 mm^2^ or US measured CSA between 10 and 13 mm^2^ plus positive nerve conduction studies. Even though the diagnosis of CTS was made accordingly to different criteria, a non-significant trend towards a higher prevalence of finger flexor tendon tenosynovitis in patients with RA and CTS compared with patients with RA without CTS was found (26.7% vs 15.8%), in line with our results. However, a comparison with US findings of idiopathic CTS was not an aim of that study; thus, a control group with idiopathic CTS was not included. Finally, in contrast to our results, in the cohort assessed by Karadag et al. a correlation between CTS and RA disease activity was not found. This might be partly explained by the different disease activity measure used (i.e. Disease Activity Score using 28 joints count vs CDAI).

Hammer et al. [21] evaluated median nerve CSA in 12 patients with rheumatic diseases (RA, *n* = 7; psoriatic arthritis, *n* = 2; ankylosing spondylitis, *n* = 1; Sjögren syndrome, *n* = 1; unspecified polyarthritis, *n* = 1) and typical CTS symptoms referred for surgical treatment and observed a higher CSA of the median nerve in this cohort compared with patients with RA without symptoms of CTS and healthy controls. However, their scanning protocol did not include the assessment of inflammatory findings at carpal tunnel level.

The different sonographic spectrum of CTS in RA patients and idiopathic CTS reflects the different pathophysiology of these two conditions. Idiopathic CTS is usually caused by a slow process of mechanical compression with consequent development of progressive morphostructural changes of the median nerve. Conversely in CTS secondary to inflammatory conditions such as RA, synovial inflammation may induce symptomatic compression of the nerve in a shorter interval of time, not sufficient to cause a significant median nerve swelling.

The results of our study support the hypothesis that the CSA of the median nerve has a weak diagnostic potential in inflammatory CTS. The value of a significant increase of median nerve CSA seems to be more relevant as a useful biomarker in the area of pathophysiology and to evaluate the extent of anatomical damage.

The detection of US inflammatory findings at carpal tunnel level, such as radio-carpal joint synovitis or flexor tendons tenosynovitis, might have important implication also from a therapeutic point of view. The presence of tenosynovitis or synovitis of the wrist might suggest a local steroid injection, especially when symptoms are present. US capability of identifying an inflammatory CTS may be helpful to explore the indication to perform an US-guided steroid injection in the target area [40]. Thus, being flexor tendons tenosynovitis and/or radio-carpal joint synovitis present in 39.1% of the wrists in our cohort, performing US examination should always be considered in RA patients with CTS symptoms.

CTS may be an early, and sometimes the first, manifestation of RA [41], and it is usually managed in non-rheumatologic setting. According to a recent retrospective case-control study, CTS was highlighted as one of the conditions more frequently encountered by the general practitioner in patients who were going to develop RA in the following 2 years, with an odds ratio of 2.96 [42].

Therefore, in patients with CTS, differently from electrophysiology, US can provide clues prompting a rheumatology referral in case of prominent inflammatory findings at carpal tunnel level. The predictive value of tenosynovitis of the finger flexor tendons of the hand for future development of RA has been also confirmed by magnetic resonance imaging (MRI) in patients with recent onset arthritic symptoms [43–45].

The main limitations of the present study were the small number of patients enrolled and its monocentric nature. Furthermore, as recommended we adopted clinical diagnosis as reference standard [5]; however, this might have led to an overestimation of CTS prevalence in RA patients, where an accurate distinction from arthritic symptoms may not be easy. Nevertheless, we found a good correlation between CTS clinical diagnosis and neuropathic pain assessed with painDETECT questionnaire. Finally, the disease activity of RA patients was calculated using the CDAI. However, the single components of this score (i.e. SJC) were not systematically registered. The lack of these data might have affected the evaluation of the correlation between the US findings and the clinical features (i.e. joint tenderness and/or swelling), which would have been of interest especially at wrist level. However, the main aim of the current study was to compare the US findings at carpal tunnel level in patients with RA and patients with idiopathic CTS. Indeed, the prevalence of synovitis and tenosynovitis at wrist level was evaluated on US and, as reported, was significantly higher in the RA patients than in the patients with idiopathic CTS.

In conclusion, the sonographic spectrum of CTS in RA patients is characterized by an inflammatory pattern, defined by the presence of finger flexor tendons tenosynovitis and/or radio-carpal joint synovitis. Conversely, a marked median nerve swelling is the dominant feature in idiopathic CTS. Intraneural PD signal is a frequent finding in both conditions.

## Data Availability

Not available because of ongoing research.
